# Generation of a Novel *In Vitro* Model to Study Endothelial Dysfunction from Atherothrombotic Specimens

**DOI:** 10.1007/s10557-021-07151-9

**Published:** 2021-02-20

**Authors:** Susan Gallogly, Takeshi Fujisawa, John D. Hung, Mairi Brittan, Elizabeth M. Skinner, Andrew J. Mitchell, Claire Medine, Neus Luque, Erika Zodda, Marta Cascante, Patrick W. Hadoke, Nicholas L. Mills, Olga Tura-Ceide

**Affiliations:** 1 BHF Centre for Cardiovascular Science, University of Edinburgh, Edinburgh, UK; 2 BHF Centre for Vascular Regeneration, University of Edinburgh, Edinburgh, UK; 3 Servei de Pneumologia, Hospital Universitari de Girona Dr. Josep Trueta, Girona Biomedical Research Institute-IDIBGI, Girona, Spain; 4 Department of Biochemistry and Molecular Biomedicine and Institute of Biomedicine-IBUB, Faculty of Biology, Universitat de Barcelona, Barcelona, Spain; 5 CIBER of Hepatic and Digestive Diseases (CIBEREHD) and Metabolomics Node at Spanish National Bioinformatics Institute (INB-ISCIII-ES-ELIXIR), Institute of Health Carlos III (ISCIII), Madrid, Spain; 6 Biomedical Research Networking Center on Respiratory Diseases (CIBERES), Madrid, Spain; 7 Department of Pulmonary Medicine, Hospital Clínic-Institut d’InvestigacionsBiomèdiques August Pi i Sunyer (IDIBAPS), University of Barcelona, Barcelona, Spain

**Keywords:** Percutaneous coronary intervention, Acute myocardial infarction, Endothelium, Endothelial dysfunction, Translational medicine

## Abstract

**Purpose:**

Endothelial dysfunction is central to the pathogenesis of acute coronary syndrome. The study of diseased endothelium is very challenging due to inherent difficulties in isolating endothelial cells from the coronary vascular bed. We sought to isolate and characterise coronary endothelial cells from patients undergoing thrombectomy for myocardial infarction to develop a patient-specific in vitro model of endothelial dysfunction.

**Methods:**

In a prospective cohort study, 49 patients underwent percutaneous coronary intervention with thrombus aspiration. Specimens were cultured, and coronary endothelial outgrowth (CEO) cells were isolated. CEO cells, endothelial cells isolated from peripheral blood, explanted coronary arteries, and umbilical veins were phenotyped and assessed functionally in vitro and in vivo.

**Results:**

CEO cells were obtained from 27/37 (73%) atherothrombotic specimens and gave rise to cells with cobblestone morphology expressing CD146 (94 ± 6%), CD31 (87 ± 14%), and von Willebrand factor (100 ± 1%). Proliferation of CEO cells was impaired compared to both coronary artery and umbilical vein endothelial cells (population doubling time, 2.5 ± 1.0 versus 1.6 ± 0.3 and 1.2 ± 0.3 days, respectively). Cell migration was also reduced compared to umbilical vein endothelial cells (29 ± 20% versus 85±19%). Importantly, unlike control endothelial cells, dysfunctional CEO cells did not incorporate into new vessels or promote angiogenesis in vivo.

**Conclusions:**

CEO cells can be reliably isolated and cultured from thrombectomy specimens in patients with acute coronary syndrome. Compared to controls, patient-derived coronary endothelial cells had impaired capacity to proliferate, migrate, and contribute to angiogenesis. CEO cells could be used to identify novel therapeutic targets to enhance endothelial function and prevent acute coronary syndromes.

**Supplementary Information:**

The online version contains supplementary material available at 10.1007/s10557-021-07151-9.

## Introduction

Atherothrombosis is characterised by atherosclerotic plaque rupture with thrombus formation and is the major cause of acute coronary syndromes and cardiovascular death. Disruption of the endothelial cell monolayer has been implicated in the onset [1], progression [2], and clinical manifestations [3] of atherothrombosis. This monolayer acts as a non-adhesive surface for platelets and leucocytes and produces important factors in the regulation of inflammation, thrombosis, and blood flow [4]. It is now widely recognised that a variety of cardiovascular risk factors including cigarette smoking and hypercholesterolemia cause endothelial dysfunction [5].

Our understanding of the cellular mechanisms of endothelial dysfunction in coronary heart disease has been hampered by our inability to isolate and study endothelial cells from the coronary circulation. Most of our observations have been based on an assessment of blood flow in the forearm [6, 7] or from measurement of surrogate biomarkers in plasma, such as von Willebrand factor (vWF) and soluble thrombomodulin [8]. Whilst the number of circulating endothelial cells can be quantified in blood [9, 10] and novel methods have been developed to isolate endothelial cells from superficial veins and arteries [11], our understanding of the cellular mechanisms of endothelial dysfunction in acute coronary syndrome is largely inferred from the study of endothelial cells from more readily available vascular beds. The study of late endothelial outgrowth cells (EOCs) from peripheral blood [12, 13] whose origin is controversial [14], or commercially acquired coronary artery endothelial cells from vascular beds of unknown health, or umbilical vein endothelial cells [15] from vessels that do not develop atherosclerosis, provides important but limited insights into the pathogenesis of coronary artery disease [16, 17].

We therefore sought to isolate and characterise coronary endothelial cells directly from thrombectomy specimens in patients undergoing treatment for acute myocardial infarction in order to generate a patient-specific in vitro model of endothelial dysfunction in coronary heart disease. We hypothesised that coronary endothelial cells would have impaired function compared to endothelial cells derived from other vascular beds.

## Methods

An expanded Methods section is available in the Online Data Supplement

### Study Population

Coronary atherothrombotic specimens (*n* = 49) were obtained from patients receiving percutaneous coronary intervention (PCI) with thrombectomy for the treatment of acute ST-segment elevation myocardial infarction (STEMI). Venous blood (*n* = 3) was obtained from patients with prior myocardial infarction. The study protocol was granted by the East of Scotland Research Ethics Service REC1 (15/ES/0094). The project was also approved by the South East Scotland BioResource Scientific Review Committee (SR019). All samples were collected with written informed consent of the participants.

### Patient and Public Involvement statement:

Patients and the public were not involved in the design, reporting, or dissemination of the findings from this study.

### Cell Isolation

Specimens were washed with phosphate buffered saline (PBS) and manually disaggregated. Tissue explants were seeded into collagen-I coated 6-well plates and maintained under standard cell culture conditions. After 24 h, tissue explants, non-adherent cells, and debris were aspirated. Medium was changed every other day until the first passage of coronary endothelial outgrowth (CEO) cells. Late EOCs were derived by seeding the mononuclear cell fraction of peripheral blood onto collagen-I coated 6-well plates as previously described [12, 13]. Human coronary artery endothelial cells (HCAECs) and human umbilical vein endothelial cells (HUVECs) were obtained commercially from single and/or pooled donors. All cultured endothelial cells were maintained under identical conditions.

### Cellular Characterisation of Atherothrombotic Specimens

Atherothrombotic specimens were formalin-fixed and paraffin-embedded. For histology, tissue sections (5 μm) were stained using haematoxylin and eosin or the Carstairs’ method for fibrin and platelets [18]. For immunohistochemistry, tissue sections are stained with monoclonal antibodies and visualised using 3,3'-diaminobenzidine (DAB) (Online Table 1). For flow cytometry, freshly isolated whole atherothrombotic specimens were washed and manually disaggregated. A single cell homogenate is achieved using collagenase, and specimens are directly stained for cell surface antigen positivity (Online Table 2).

### Phenotypic Characterisation of Endothelial Cells

For immunocytochemistry, cells are fixed in 4% paraformaldehyde and stained using human monoclonal antibodies and fluorochrome-conjugated secondary antibodies (Online Table 2). Cells are phenotyped at each passage for flow cytometric positivity of endothelial cell surface antigens, endothelial cytoplasmic antigens, and leucocyte antigens (Online Tables 2 and 3). For uptake of acetylated low-density lipoprotein (LDL), confluent monolayers of endothelial cells were incubated for 4 h with 1,1-dioctadecyl-3,3,3tetramethylindocarbocyanine (DIL)-labelled acetylated LDL. For quantification of western blot VEGF protein levels, cells were lysed and incubated with anti-human VEGF antibody following manufacturer’s guidelines.

### In Vitro Functional Characterisation of Endothelial Cells

For growth kinetics, population doubling times (PDT) were calculated according to the equation PDT = Log^2^ (C_*h*_/C_*s*_)/*t*, where C_*h*_ is the cell number harvested, C_*s*_ is the cell number seeded, and *t* is the time interval [12]. PDT data from the first 9 cell passages were averaged for all samples. Cumulative population doubling levels (CPDL) were the sum of all population doublings. Cell adhesion was assessed by seeding cells into a 6-well collagen I-coated plate for 30 min as previously described [19]. Plates were washed, and attachment within a defined region of each well was quantified and expressed as a percentage of seeded cell number. For the wound migration assay, cells were grown to confluence and rendered quiescent by incubation with serum-free medium for 24 h as previously described [18]. The endothelial cell monolayer was wounded by a linear vertical stroke across the diameter of the well using a P1000 pipette tip. To quantify migration, the width of the wound was visualised at the start time (0 h), and the area of wound closure across the vertical stroke at 24 h was quantified and expressed as a percentage of wound closure. To assess angiogenic potential, cells were seeded into a 48-well plate pre-coated with Matrigel™ basement membrane matrix, and tube-like structures were quantified at 24 h. Nitrite concentrations were measured by Griess Reagent (ab234044) assay following manufacturer’s instructions. Glucose consumption as a measurement of cell metabolism was determined by spectrophotometry (COBAS Mira Plus, Horiba ABX) from cell culture media (48 h) by monitoring the production of NAD(P) H in the specific reaction at 340 nm wavelength.

### In Vivo Angiogenesis Assay

Animal experiments to evaluate in vivo neovascularisation were performed in NOD-SCID gamma mice (NOD.Cg-PrkdcscidIl2rgtm1WjI/SzJ) aged 10–12 weeks (*n* = 28), in accordance with the British Home Office Animals (Scientific Procedures) Act 1986. Mice were anaesthetised intraperitoneally with Domitor containing medetomidine and vetalar containing ketamine; both were administered at 0.1 ml per 10 g body weight. Upon sedation, an analgesic, vetergesic containing buprenorphine was administered subcutaneously at 0.1 mg per kg body weight. Sterile sponges embedded with growth factor reduced (GFR)-Matrigel™ (vehicle control) and GFR-Matrigel™ with endothelial cells (CEO, EOC and HUVECs) were subcutaneously implanted for 21 days [20] after which mice were sacrificed by cervical dislocation. Murine sponges were formalin fixed and 4 μm sections stained with haematoxylin and eosin. Angiogenesis was assessed using the Chalkley count method [13, 20, 21]. Host vessels with human cells were identified using human-specific monoclonal antibodies to endothelial cell antigens as previously described [19], and the percentage of human-specific vessels were quantified.

### Statistical Analysis

Data are shown as mean ± standard deviation. Independent samples were analysed using the unpaired Student’s *t* test. More than two groups were compared using repeated measure or one-way analysis of variance (ANOVA) with Bonferroni post-tests where appropriate. Categorical data were compared using the chi-squared with Fisher’s exact test. Paired Student’s *t* tests were used to compare Chalkley counts between vehicle control- and cell-infiltrated sponges. Statistical significance was assumed if a null hypothesis could be rejected at *P* ≤ 0.05.

## Results

Coronary atherothrombotic specimens (*n* = 49) are collected from consecutive patients with acute myocardial infarction with specimens fixed for histology (*n* = 8), flow cytometry (*n* = 4), or manually disaggregated for cell culture (*n* = 37) (Online Fig. 1). Patients are 62 ± 12 years old (76% male) with typical risk factors for acute coronary syndrome (Online Table 4).

### Atherothrombotic Specimens

**Fig. 1:**
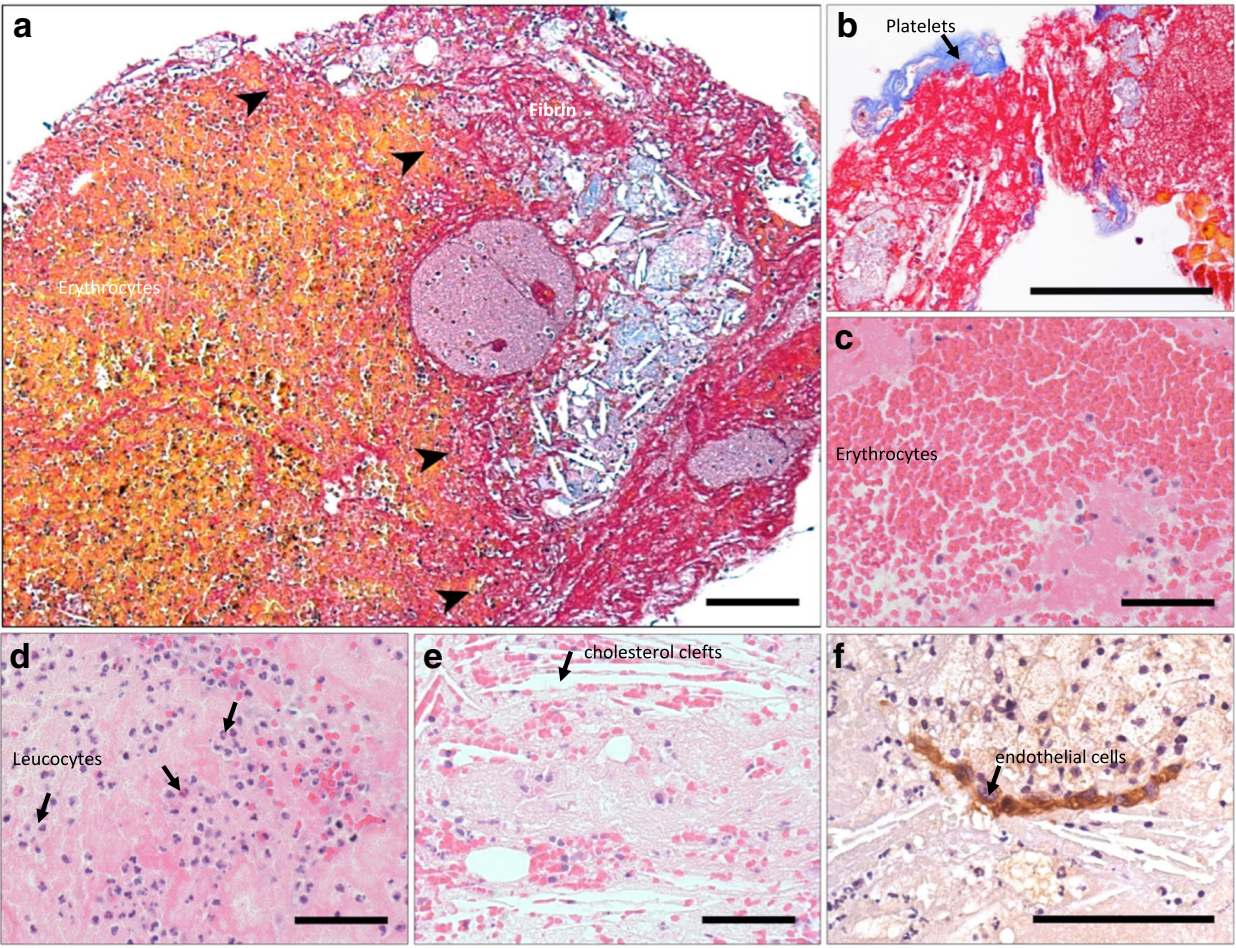
Coronary atherothrombotic specimens from patients undergoing treatment for ST-segment elevation myocardial infarction. The border zone between cholesterol cleft rich atheroma and thrombus is evident (arrows) with Carstairs staining rendering erythrocytes, yellow, and fibrin, pink (**a**). Atherothrombotic specimens were composed of platelets (blue) (**b**), erythrocytes (**c**), leucocytes (**d**), cholesterol clefts (**e**), and CD146^+^ endothelial cells (**f**) encased in fibrin. Scale bars 50 μm

Histological examination of atherothrombotic specimens identifies platelets, erythrocytes, leucocytes, and fibrin with atherosclerotic plaque containing cholesterol clefts (Fig. 1a–e**)**. Clusters of CD146^+^ cells are visualised (Fig. 1f), but no microvessels are found. Flow cytometry analysis identified CD146^+^and CD31^+^endothelial cells in disaggregated atherothrombotic specimens (CD45^-^CD42a^-^CD146^+^ = 0.18 ± 0.36% and CD45^-^CD42a^-^CD31^+^ = 0.11 ± 0.12% of viable cells) (*n* = 4) (Online Fig. 2).

### Isolation and Characterisation of Coronary Endothelial Outgrowth Cells

**Fig. 2:**
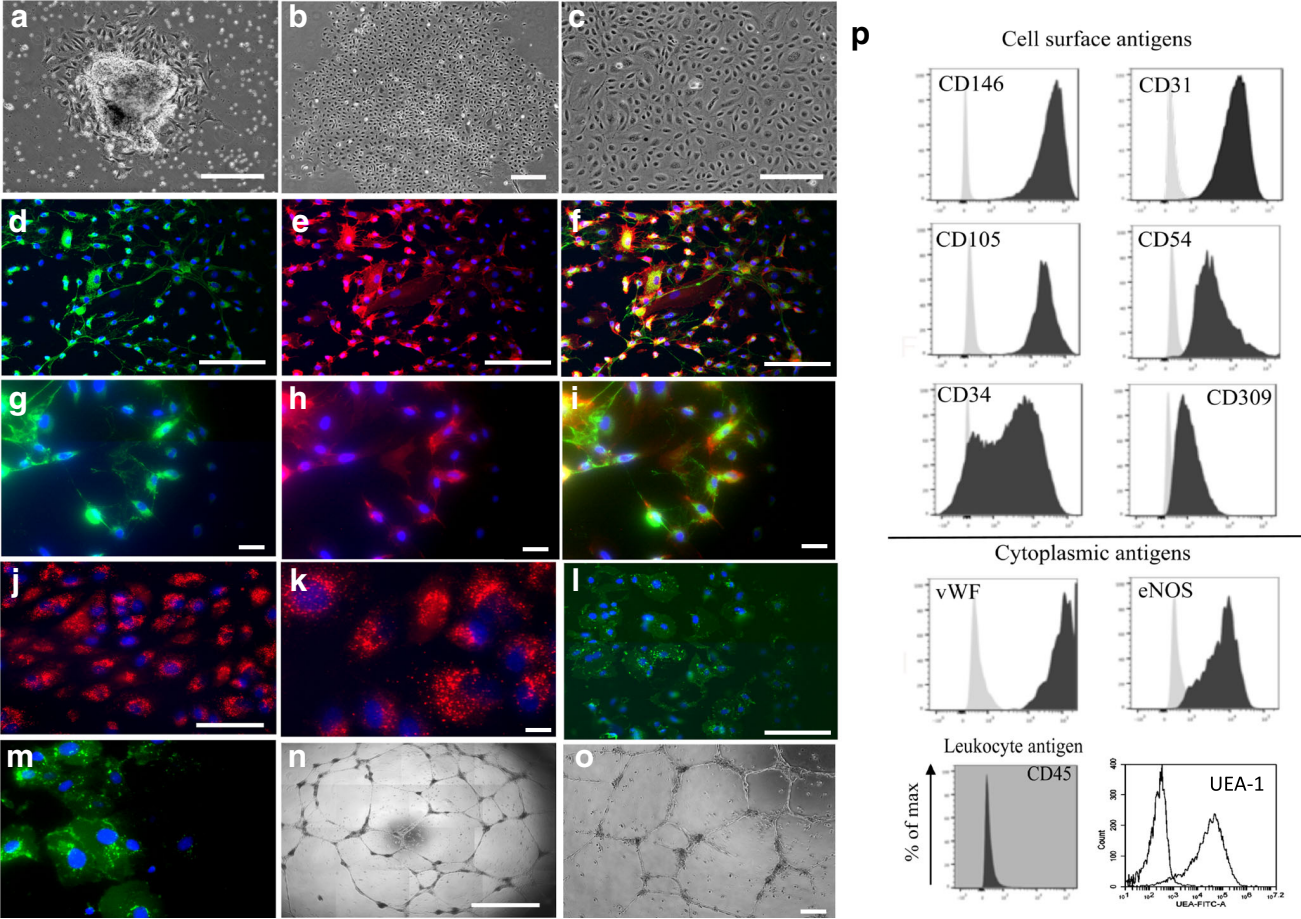
Outgrowth and characterisation of coronary endothelial outgrowth cells from atherothrombotic specimens. Outgrowth of coronary endothelial cells from dissected atherothrombotic specimen at 24 hours (**a**), colony formation at 7 days (**b**), and confluent cobblestone morphology at first passage (**c**). Cells stain positive for cytoplasmic expression of granular von Willebrand factor indicative of Weibel–Palade bodies (pink) and cell surface expression of CD31 (green) with 4*'*,6-diamidino-2-phenylindole (DAPI) as a nuclei counterstain (blue) (**d**). Uptake of fluorescent labelled Dil-acetylated-LDL (red) with DAPI as a counterstain (**e**) and tube-like structure formation on Matrigel^TM^(**f**). Scale bar 100 μm. Coronary endothelial outgrowth cells strongly express cell surface antigens CD146, CD31, CD105, and CD54 and cytoplasmic antigens von Willebrand factor (vWF) and endothelial nitric oxide synthase (eNOS), but do not express the pan leucocyte antigen CD45. Unstained (grey) and stained cells (black) (**g**)

Outgrowth from atherothrombotic specimens is obtained from 27/37 (73%) specimens (Online Table 5). Outgrowth was isolated more frequently in atherothrombotic specimens from the right coronary artery (21/23, 91%) than the left anterior descending artery (4/12, 33%). Colonies emerged after 2–15 days in culture forming a median of 2 colonies per specimen (interquartile range of 6 colonies) (Fig. 2a and b). Cells are first passaged after 21 days (range 15–28 days) and continued to proliferate to form a confluent monolayer (Fig. 2c) with 21/27(78%) cell lines proliferating for multiple passages (range 6–17).

Outgrowth cells had typical endothelial cell morphology with a centrally located circular nucleus. Cells stained for vWF and had uniform cell surface CD31 expression (Fig. 2d–l). Cells took up fluorescent-labelled acetylated-LDL (Fig. 2j, k) stained positive for *Ulex europaeus* lectin (Fig. 2l, m) and formed tube-like structures on a Matrigel^TM^ membrane matrix (Fig. 2n, o) and were defined as coronary endothelial outgrowth (CEO) cells. These cells were strongly positive for endothelial cell surface and cytoplasmic antigens: CD146 (90%), CD31 (87,1%), CD105 (93,4%), intracellular adhesion molecule-1(ICAM-1) (81,8%), vWF (99,9%), UEA-1 (94%), and endothelial nitric oxide synthase (eNOS) (93,4%). A proportion of CEO cells were positive for CD34 (63,5%), CD309 (kinase domain receptor, KDR) (76,8%), and CD133 (33,5%). Whilst the majority of endothelial antigens was unchanged during culture, CD34 and CD133 positivity diminished (*P* < 0.01) and CD105 increased (*P* < 0.01). Positivity of the non-endothelial surface antigen CD45 remains low (< 3%), and cells are negative for αSMA (Online Table 6**,** Fig. 2p, and Online Fig. 3).

### Phenotypic and Functional Comparison of Endothelial Cells

#### Antigen Expression

CEO cells had a similar phenotype to control endothelial cells (EOCs, HCAECs, and HUVECs) during early passage (1-4), although CEO cells had lower CD45 positivity compared with EOCs (*P* < 0.01; Online Table 7). At later passage (9-12), CEO cells presented lower positivity of CD34 and higher levels of CD309 and CD133 in comparison with HCAECs (*P* < 0.05, *P* < 0.001, and *P* < 0.001, respectively).

#### Growth Kinetics and Glucose Consumption

**Fig. 3:**
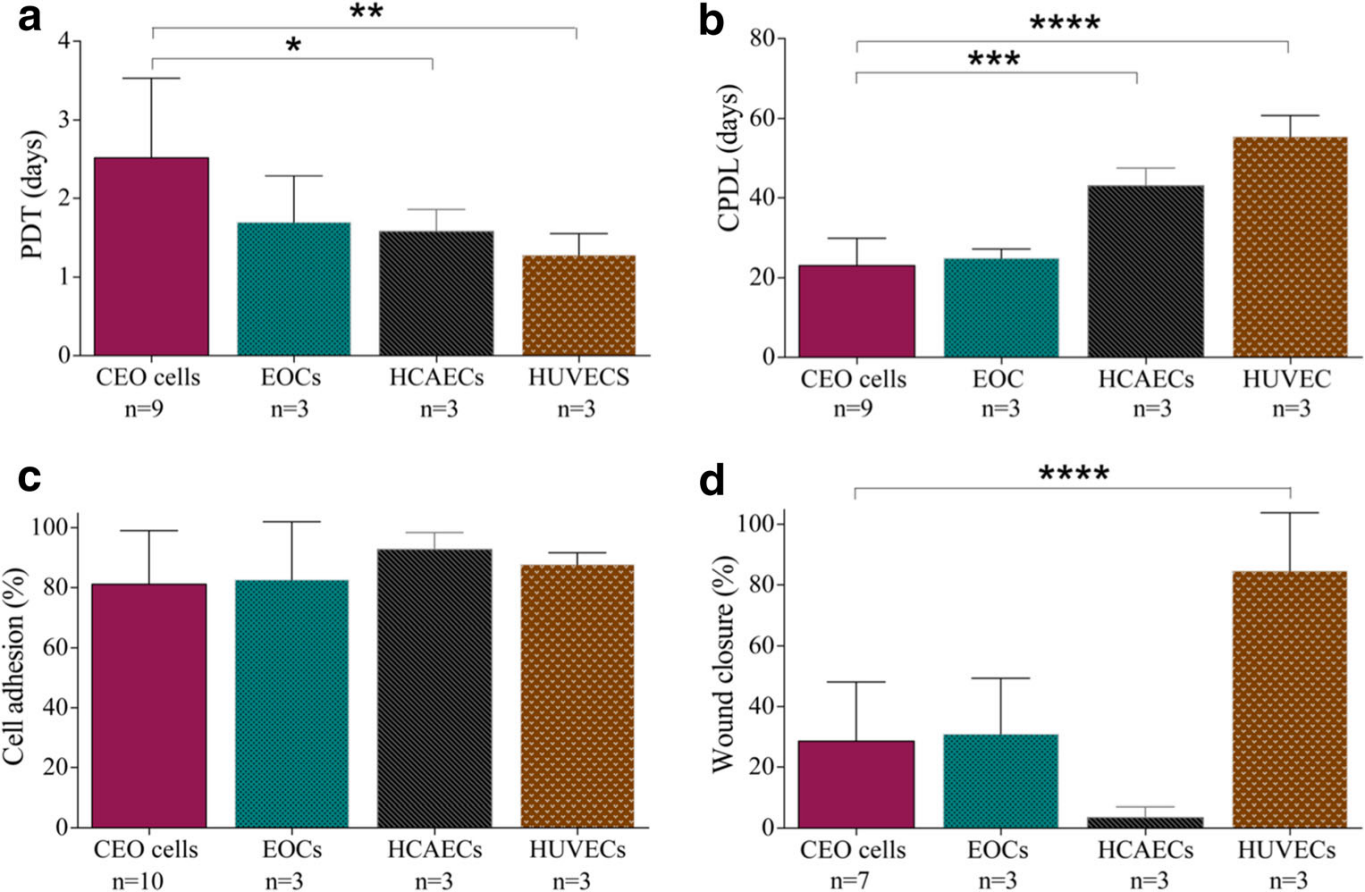
Proliferation, adhesion, and wound migration of coronary endothelial outgrowth cells in vitro. Coronary endothelial outgrowth (CEO) cells had a higher population doubling time (PDT) during the first 9 passage in comparison to human coronary artery endothelial cells (HCAECs) and human umbilical vein endothelial cells (HUVECs) (one-way analysis of variance *P* = 0.017; Bonferroni post-test *P < 0.05 and **P < 0.01, respectively. *n* = 3–9) (**a**) and a lower cumulative population doubling level (CPDL) when maintained long term in culture (*P* < 0.0001; Bonferroni post-test ***P < 0.001 and ****P < 0.0001, respectively. *n* = 3–9) (**b**). CEO cells had a similar capacity to adhere to a collagen substrate in vitro (*P* = 0.662. *n* = 3–10) (**c**), but following the infliction of a linear wound in the endothelial cell monolayer, CEO cells had a reduced capacity to migrate in vitro in comparison to HUVECs (*P* = 0.008; Bonferroni post-test ****P < 0.0001. *n* = 3–7) (**d**)

Proliferative activity is lower in CEO cells (population doubling time [PDT] = 2.5 ± 1.0 days) compared to healthy HCAECs (PDT = 1.6 ± 0.3 days; *P* < 0.05) and HUVECs (PDT = 1.2 ± 0.3 days; *P* < 0.01) (*P* = 0.017) (Fig. 3a). CEO cells had a lower cumulative population doubling level (23.0 ± 6.8 days) during prolonged culture (8 passages) than HCAECs (43.1 ± 2.1 days) and HUVECs (55.5 ± 5.3 days) (*P* < 0.001 and *P* < 0.0001, respectively) (*P* < 0.0001) (Fig. 3b). Metabolic measurement of percentage of glucose consumption as another indicator of cellular activity is shown to be significantly reduced in CEO cells compared to healthy HCAECs (Online Fig. 4**)**.

### In Vitro Adhesion, Migration, Angiogenesis Assay, and Nitrite Concentration

CEO cells had a similar capacity to adhere to a collagen substrate (81 ± 18%) than EOCs (83 ± 19%), HCAECs (93 ± 6%), and HUVECs (88 ± 4%) (*P* = 0.662) (Fig. 3c). The migratory capacity of CEO cells across a linear wound is reduced compared to HUVECs (29 ± 20% *versus* 85 ± 19% wound closure; *P* < 0.0001), but did not differ compared to EOCs (31 ± 19%) and HCAECs (4 ± 4%) (*P* = 0.008) (Fig. 3d**)**. CEO cells formed a similar number of tube-like structures on Matrigel^TM^ (20 ± 30 tubules) compared to EOC (20 ± 11 tubules), HCAECs (26 ± 6 tubules), and HUVECs (44 ± 58 tubules) (*P* = 0.415). Additionally, CEO cells had similar VEGF protein levels compared to HCAECs measured by western blot and similar nitrite concentration levels than control HUVECs and HCAECs (Online Fig. 4**).**

### In Vivo Angiogenesis Assay

**Fig. 4:**
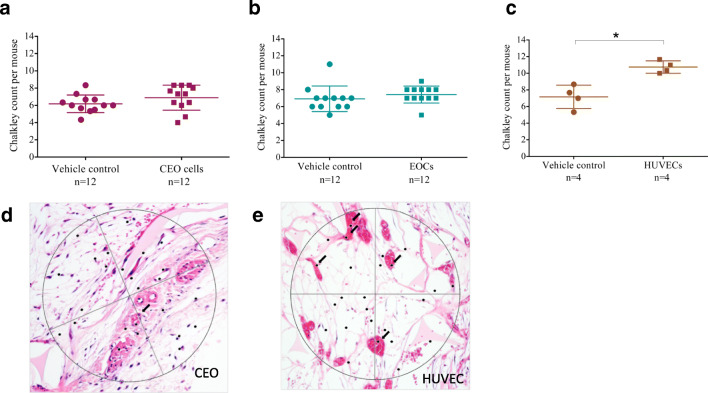
Angiogenic potential of coronary endothelial outgrowth cells in vivo. Sponges embedded with growth factor reduced (GFR)-Matrigel^TM^ (vehicle control) and GFR-Matrigel^TM^ with coronary endothelial outgrowth (CEO) cells (**a**), late endothelial outgrowth cells (EOCs) (**b**), or human umbilical vein endothelial cells (HUVECs) (**c**) were subcutaneously implanted into male NOD-SCID gamma mice. CEO cells and EOCs did not increase blood vessel number compared to vehicle controls (paired *t* test *P* = 0.207 and *P* = 0.365, respectively. *n* = 12), whereas HUVECs did increase vessel number (paired *t* test *P* = 0.030. *n* = 4). Light microscopy of haematoxylin/eosin stained sponge 4-μm sections from CEO impregnated sponge (**d**) and HUVEC impregnated sponge (**e**) (black arrows identify vessels)

**Fig. 5:**
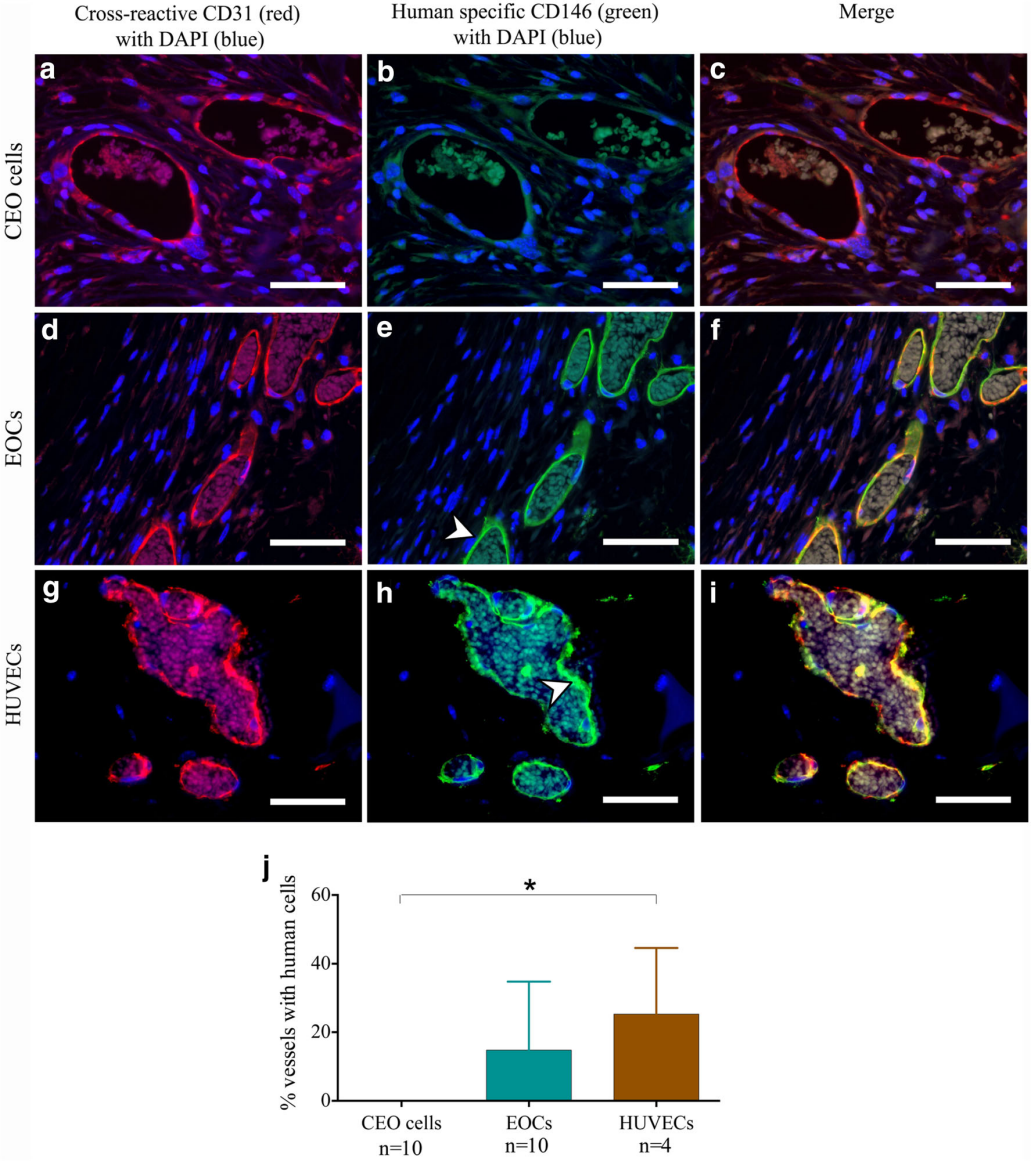
Coronary endothelial outgrowth cells do not incorporate into murine vessels in vivo. Immunohistochemistry of subcutaneously implanted sponges embedded with CEO cells (**a**–**c**), EOCs (**d**–**f**), and HUVECs (**g**–**i**). Sections were stained with antibodies cross-reactive to both mouse and human antigens (red) or specific to human antigens (green) using 4*'*,6-diamidino-2-phenylindole (DAPI) as a counterstain. Auto-fluorescent erythrocytes are presence in vessel lumen demonstrates the vessels were contiguous with the host circulation. Scale bars 50 μm. In contrast to late endothelial outgrowth cells (EOCs) and human umbilical vein endothelial cells (HUVECs), coronary endothelial outgrowth (CEO) cells did not incorporate into murine vessel (**j**) (one-way analysis of variance *P* = 0.019; Bonferroni post-test *P < 0.05 for CEO cells *versus* HUVECs. *n* = 4–10)

Implantation of CEO cells into a subcutaneous sponge did not alter vessel density compared to vehicle control (Chalkley count 7 ± 1 *versus* 6 ± 1, *P* = 0.207) (Fig. 4a), nor did CEO cells incorporate into vascular structures (Fig. 5a–c and j). HUVECs, but not EOCs, increased vessel density compared to vehicle control (11 ± 1 *versus* 7 ± 1, *P* = 0.030 and 7 ± 1 *versus* 7 ± 2 *P* = 0.365 respectively; Fig. 4b and c), and both are found to incorporate into murine vessels (HUVECs: 25±19%; EOCs: 25 ± 19% vessels with human cells) (*P* = 0.019) (Fig. 5d–i and j).

## Discussion

Endothelial dysfunction is key to the pathogenesis of acute coronary syndrome. However, our understanding of the role of the coronary endothelium is based largely on the study of endothelial cells derived from more readily available vascular beds. Here, we report the first successful expansion of coronary endothelial cells from coronary atherothrombotic specimens obtained from patients undergoing treatment for acute myocardial infarction. We systematically compared the phenotype and function of these cells with endothelial outgrowth progenitor cells isolated from the peripheral circulation and with mature human coronary artery cells and umbilical vein endothelial cells. This study demonstrated that CEO cells are phenotypically similar to control mature endothelial cells but have marked diseased-specific dysfunctional characteristics both in vitro and in vivo*.* Our results indicate that atherothrombotic specimens are a viable and important sources of endothelial cells to understand the role and the insights of endothelial dysfunction in the pathogenesis of coronary artery disease.

Studied atherothrombotic specimens were heterogeneous and rich in erythrocytes, leucocytes, fibrin, and cholesterol clefts as previously reported [22, 23]. We found isolated endothelial cells and buried fibrous caps, although the number of CD146^+^ and CD31^+^cells in digested specimens was low (< 1%) which is consistent with previous observations [24]. We derived outgrowth from manually dissected whole atherothrombotic specimens without cell selection based on endothelial cell surface markers, and therefore, we carefully assessed our outgrowth for endothelial phenotypic markers and functional characteristics. CEO cells were positive for variety of mature endothelial cell markers, incorporated acetylated LDL, and formed tube-like structures on Matrigel^TM^ confirming an endothelial cell identity. Furthermore, the proportion of outgrowth cells that were positive with the pan-leucocyte marker CD45 was low (< 4%) suggesting minimal contamination from inflammatory cells.

To our knowledge, we report the first attempt to isolate, expand, and characterise endothelial cells from atherothrombotic specimens. One previous study obtained endothelial cells from coronary guidewires using CD146 immunomagnetic beads [25]. However, they were unable to expand these cells in vitro, and limited characterisation was performed. Although the authors speculate that they were unable to expand cells due to endothelial dysfunction, it is as likely that expansion failed due to the very low yield of cells from the coronary guidewire. Other methods involving wire biopsies of the superficial veins and arteries of the forearm have been more successful in the isolation and expansion of endothelial cells in culture [11, 26, 27], but these beds are largely protected from atherosclerosis. As such, we suggest that CEO cells obtained from atherothrombotic specimens, the most representative and relevant endothelial cells to study the pathogenesis of atherosclerosis.

Compared with commercially available control endothelial cells from human cadaveric coronary arteries and human umbilical veins, CEO cells had lower rates of proliferation and glucose consumption. Furthermore, CEO cells had a reduced migratory and angiogenic capacity compared with HUVECs and were unable to incorporate into new vascular beds in our experimental model of angiogenesis. Given other aspects of endothelial function, such as vasodilatation and fibrinolysis, are impaired in patients with coronary artery disease [5, 28], this result is not unexpected. As CEO cells were isolated from ruptured plaques during an acute myocardial infarction, it is likely that they have experienced oxidative stress and ischemic injury affecting their function. As these differences persisted in CEOs following multiple passages and expansion in optimal culture conditions, our results indicate there is an inherent difference in the function of endothelial cells derived from coronary atherosclerotic plaques.

Further work is required to understand the mechanistic pathways that influence the function of coronary endothelial outgrowth cells, including the role of clinical features, cellular metabolism, inflammation, and *shear stress*. This research could help identify new targets for drug discovery beyond established pathways such as nitric oxide biosynthesis, fibrinolysis, and platelet aggregation, which could link coronary endothelial dysfunction to acute atherothrombosis.

## Limitations

It was not possible to obtain coronary artery endothelial cells from age-matched controls given the invasive nature of the thrombectomy procedure, and therefore, we cannot definitively conclude that functional differences of CEO cells are a cause or consequence of atherosclerosis, coronary heart disease, or age. Furthermore, whilst all cells were cultured in identical conditions and evaluated as the same passage, we cannot completely exclude that the method used to isolated cells could account for some of the differences observed. However, we included multiple controls including endothelial outgrowth cells from patients with coronary heart disease and coronary artery endothelial cells isolated from cadaveric hearts.

## Summary

In conclusion, this study reports a novel method to isolate and expand coronary endothelial cells from thrombectomy specimens, harvested during primary percutaneous coronary intervention for acute ST-segment elevation myocardial infarction. Our results show that these cells are dysfunctional with an impaired capacity to proliferate, migrate, and contribute to angiogenesis, compared to healthy endothelial cells isolated from different vascular beds. This novel approach could have an impact on clinical practice as they may be used as tools to identify and test novel therapeutic targets and molecular pathways to correct endothelial function and prevent acute coronary syndrome.

## Supplementary Information

ESM 1 (DOCX 1561 kb)

## Data Availability

All data relevant to the study are included in the article or uploaded as supplementary information. This data was also presented as an abstract at https://www.atherosclerosisjournal.com/article/S0021-9150(15)00414-1/fulltext.

## Non-standard Abbreviations and Acronyms

ANOVA: Analysis of variance
APC: Allophycocyanin
CEO: Coronary endothelial outgrowth
CPDL: Cumulative population doubling level
DAB: 3,3’-Diaminobenzidine
DAPI: 4',6-Diamidino-2-phenylindole
DIL: 1,1′-Dioctadecyl-3,3,3′tetramethylindocarbocyanine
EDTA: Ethylenediaminetetraacetic acid
eNOS: Endothelial nitric oxide synthase
EOCs: Endothelial outgrowth cells
FBS: Foetal bovine serum
FITC: Fluorescein isothiocyanate
GFR: Growth factor reduced
HCAECs: Human coronary artery endothelial cells
HUVECs: Human umbilical vein endothelial cells
KDR: Kinase domain receptor
LDL: Low-density lipoprotein
NGS: Normal goat serum
PBS: Phosphate buffered saline
PCI: Percutaneous coronary intervention
PDT: Population doubling time
PE: Phycoerythrin
PFA: Paraformaldehyde
STEMI: ST-segment elevation myocardial infarction
vWF: von Willebrand factor
