# Supplementary material for: Generation of a Novel In Vitro Model to Study Endothelial Dysfunction from Atherothrombotic Specimens
Source: Cardiovasc Drugs Ther. 2021 Feb 20;35(6):1281–90. doi: 10.1007/s10557-021-07151-9 (PMC8578063; doi:10.1007/s10557-021-07151-9)
Supplement: Supplementary file 1 — (DOCX 1561 kb) [file 10557_2021_7151_MOESM1_ESM.docx]

**SUPPLEMENTAL MATERIAL**

**Generation of a novel in vitro model to study endothelial dysfunction from atherothrombotic specimens.**

**Short title:** Gallogly - Coronary endothelial cells from thrombectomy

Susan Gallogly PhD^1,2^, Takeshi Fujisawa PhD^1,2^, John D Hung MD^1^, Mairi Brittan PhD^1,2^,

Elizabeth M Skinner PhD^1,2^, Andrew J Mitchell MD^1^, Claire Medine PhD^1,2^, Neus Luque^3^, Erika Zodda^4^, Marta Cascante^4,5^, Patrick W Hadoke PhD^1^_,_ Nicholas L Mills MD PhD^1,2*^,

Olga Tura-Ceide PhD^3,6,7 *^

^1^ BHF Centre for Cardiovascular Science, University of Edinburgh, Edinburgh, UK

^2^ BHF Centre for Vascular Regeneration, University of Edinburgh, Edinburgh, UK

^3^ Servei de Pneumologia, Hospital Universitari de Girona Dr. Josep Trueta, Girona Biomedical Research Institute-IDIBGI, Girona, Spain.

^4^Department of Biochemistry and Molecular Biomedicine and Institute of Biomedicine-IBUB, Faculty of Biology, Universitat de Barcelona, Barcelona, Spain.

^5^CIBER of Hepatic and Digestive Diseases (CIBEREHD) and Metabolomics Node at Spanish National Bioinformatics Institute (INB-ISCIII-ES- ELIXIR), Institute of Health Carlos III (ISCIII), Madrid, Spain.

^6^ Biomedical Research Networking Center on Respiratory Diseases (CIBERES), Madrid, Spain.

^7^DepartmentofPulmonary Medicine, Hospital Clínic-Institut d'Investigacions Biomèdiques August Pi i Sunyer (IDIBAPS); University of Barcelona; Barcelona, Spain.

**Word count:** 2869

**Contents:** Detailed methodology and on-line tables and figures

**DETAILED METHODS**

**Study population**

Atherothrombotic specimens from patients receiving emergency percutaneous coronary intervention and manual thrombus aspiration for acute ST-segment elevation myocardial infarction (n=49) were collected into complete EGM™-2 BulletKit™ medium (‘EGM™-2’; Cat no. CC-3162; Lonza, UK) supplemented with 10% HyClone™ foetal bovine serum (FBS) (Cat no. 12379802; Fisher Scientific, UK). Venous blood from patients with prior myocardial infarction (100ml) was drawn from the median cubital vein and placed in 8ml of 3.8% sodium citrate (n=3). All samples were processed within 24 hours and subjects provided written informed consent. The study protocol was granted by the East of Scotland Research Ethics Service REC1 (15/ES/0094). The project was also approved by the South East Scotland BioResource Scientific Review Committee (SR019) and this study was conducted in accordance with the declaration of Helsinki and institutional guidelines. All samples were collected with written informed consent of the participants.

**Cell isolation**

Endothelial cells were grown on tissue culture plastics pre-coated with type I rat-tail collagen (Cat no. 356400; BD Bioscience, UK) and maintained in EGM™-2 medium supplemented with 10% HyClone™ FBS unless otherwise stated.

Coronary endothelial outgrowth (CEO) cells were isolated from atherothrombotic specimens (n=37). Specimens were washed with phosphate buffered saline (PBS) and manually disaggregated using sterile surgical scalpels. Tissue explants were seeded into 1 well of a 6-well tissue culture plate and maintained under standard cell culture conditions. After 24 hours, tissue explants, non-adherent cells, and debris were aspirated. Medium was changed every other day until first passage. Cells were left in culture 15-28 days (dependent on proliferative activity) prior to first passage.

Late endothelial outgrowth cells (EOCs) were isolated from the mononuclear cell fraction of whole blood (100ml) by buoyant density centrifugation over Ficoll-Paque PLUS (Cat no. 17-1440-03; GE Healthcare, Sweden) (n=3). Without further cell subpopulation enrichment procedures, 10 x 10^6^ MNCs were resuspended in 2 wells of a 6-well tissue culture plate. After 24 hours, non-adherent cells were aspirated and fresh media was added [1-2]. EOCs were characterised by their ‘cobblestone’ morphology and cells were maintained for 21 days in culture prior to first passage.

Human coronary artery endothelial cells (HCAECs) (Cat. CC-2585; Lonza, UK), pooled human umbilical vein endothelial cells (HUVECs) (CC-2519; Lonza, UK and Cat. C-12203 Promocell, Germany) and single donor HUVECs (Cat. C-003-5C; Invitrogen, UK) were acquired commercially.

Cells were treated in an identical manner and experimental numbers are biological repeats for all except HCAECs, which are results from 3 independent experiments.

**Cellular characterisation of atherothrombotic specimens**

**Immunohistochemistry**

Freshly isolated whole atherothrombotic specimens were formalin-fixed and paraffin-embedded (n=8). Specimens were cut longitudinally into 5μm segments, deparaffinised with xylene and rehydrated with increasing concentrations of alcohol.

For Haematoxylin and Eosin staining, specimens were stained with haematoxylin for 5 minutes, rinsed with distilled water for 15 minutes and then stained with eosin for 1 minute. For the Carstairs’ method for fibrin and platelet staining, specimens were stained with 5% Ferric Ammonium Sulphate for 5 minutes, Mayer’s hematoxylin for 5 minutes and Picric Acid-orange G solution for 45 minutes. Slides were then washed and stained with Ponceau Fuchsin solution for 1 minute, 1% phosphotungstic acid for 2 minutes and Anilin blue solution for 10 minutes as previously described [3]. Slides were then washed in tap water and air dried overnight before coverslips were applied. All histological reagents were provided by Electron Microscopy Services (USA). For CD146 staining, epitopes were retrieved by boiling specimens in 1mM ethylenediaminetetraacetic acid (EDTA) solution for 20 minutes prior to endogenous peroxide activity quenching by 3% hydrogen peroxide. Specimens were treated with Endogenous Avidin/Biotin Blocking Kit (Cat no. Ab3387; Abcam, UK) according to the manufacturers’ instructions and non-specific antibody binding was prevented by incubating specimens with 10% normal horse serum (Cat no. S-200; Vector Laboratories, Inc., USA) in PBS. Specimens were incubated with monoclonal anti-human CD146 (Cat. Ab49492; Abcam, UK) in PBS with 2% normal horse serum overnight at 4°C, washed in PBS, and incubated with biotinylated horse anti-mouse secondary antibody (Cat no. BA-200; Vector Laboratories, Inc., USA) for 30 minutes at room temperature. The Vectastain Elite ABC Peroxidase kit (Cat no. PK-4000; Vector Laboratories, Inc., USA) was used for secondary antibody detection and visualisation occurred using 3,3'-Diaminobenzidine (DAB) as the substrate (Cat no. SK-4100; Vector Laboratories, Inc., USA).

**Flow cytometry**

Freshly isolated whole atherothrombotic specimens were washed with PBS and manually disaggregated (n=4). A single cell homogenate was achieved following enzymatic disaggregation in PBS containing 1mg/1ml type I collagenase (Cat. 17018-029; Life Technologies, UK) in a 37°C shaking water bath at 180rpm for 60 minutes, followed by passing cells through a 100μm sterile nylon mesh. Cells were recovered by centrifugation and resuspended in EGM™-2 medium. 100µl cell suspension was incubated for 30 minutes at room temperature with monoclonal antibodies conjugated to specific fluorochromes: LIVE/DEAD® Fixable Dead Cell Stains-V525, CD42a-FITC, CD45-V450, CD146-PE/Cy7 and CD31-PE (**Online Table 3**). After washing, cells were fixed and erythrocytes lysed simultaneously using BD FACS™ Lysing Solution (Cat no. 349202; BD Biosciences, UK). Samples were washed and then analysed using a BD LSRFortessa^TM^ II cell analyser. Unstained samples were used as controls. Spectral overlap and compensation was calculated and verified manually for each antibody using anti-mouse BD™ CompBead (Cat no. 552843, BD Biosciences, UK). A minimum of 200,000 events was collected for data analysis using FlowJo version 10.0.6 (TreeStar Inc., Switzerland). Endothelial cells were identified by the placement of population gates based on the unstained control. Following scatter selection and doublet discrimination, viable cells were selected by exclusion of LIVE/DEAD® Fixable Dead Cell Stain-V525. CD42a and CD45 positive cells were excluded to avoid platelets, hematopoietic cells and leucocytes and endothelial cells were identified by their expression of CD146 or CD31 (**Online Figure 2**).

**Phenotypic characterisation of endothelial cells**

**Cell viability**

After trypsinization and throughout all cell culture expansion, cell viability of all cell lines was measured by trypan blue exclusion test. Briefly, 10ul of resuspended endothelial cells were mixed with 10ul of Trypan Blue 0,4% solution prepared in 0.85% NaCl (Cat no.17-942E; Lonza, USA) to determine the percentage of cells that have clear cytoplasm (viable cells) versus cells that have blue cytoplasm (nonviable cells).

**Immunocytochemistry**

Endothelial cells (passage 3) grown on tissue culture vessels pre-coated with type I rat-tail collagen were washed twice with PBS and fixed with 4% paraformaldehyde (PFA) (Cat. PF101; FD Neutro technologies Inc., USA) for 10 minutes at room temperature. Cells were permeabilised with 0.1% Triton™ X-100 (Cat. T9284; Sigma Aldrich, UK) for 5 minutes, and then incubated with 2% normal goat serum (NGS) (Cat no. S-1000; Vector Laboratories Inc., USA) in PBS for 30 minutes to block non-specific staining. Cells were incubated overnight at 4°C with monoclonal antibodies: rabbit anti- human CD31, mouse anti-human vonWillebrand Factor (vWF) antibodies (**Online Table 1**) and isotype controls for mouse primary antibody (Cat no. 086599; Invitrogen, UK) and rabbit primary antibody (Cat no. 086199; Invitrogen, UK). Cells were washed and incubated with fluorescent-labelled secondary antibodies for 30 minutes: Alexa Fluor® 488 goat anti-rabbit or Alexa Fluor® 647 goat anti-mouse to detect CD31 and vWF, respectively (**Online Table 1**). Cells were washed in PBS, rinsed in tap water and mounted using ProLong® Gold Antifade Reagent with 4',6-diamidino-2-phenylindole (DAPI) (Cat no. P36935; Invitrogen, UK). Cover slips were applied and slides were dried at room temperature in the dark for 1 hour prior to analysis.

**Flow cytometry**

Endothelial cells (passage 3) were trypsinised from tissue culture vessels and aliquots of 1 x 10^5^ cells were resuspended in 100μl EGM™-2 medium and incubated for 30 minutes at room temperature with pre-conjugated monoclonal antibodies: CD146-PECy7, CD31-FITC, CD105-APC, CD144-PE, CD54-PE, CD34-APC/Cy7, CD309-PE, CD133-APC, CD117-PE, CD106-FITC, αSMA-AF405, UEA-1-FITC and CD45-V450 (**Online Table 3**). For cytoplasmic antigens, 1 x 10^5^ cells were permeabilised using BD Cytofix/Cytoperm™ Fixation/Permeabilisation solution (Cat no. 554714; BD Biosciences, UK) prior to incubation with monoclonal pre-conjugated antibodies: vWF-FITC and eNOS-PE (**Online Table 3**). After washing, cells were fixed using BD FACS™ Lysing Solution (Cat no. 349202; BD Biosciences, UK). Cells were washed and analysed using a BD LSRFortessa^TM^ II cell analyser. Unstained cells were used as controls. Spectral overlap and compensation was calculated and verified manually for each antibody using anti-mouse BD™ CompBead (Cat no. 552843, BD Biosciences, UK). A minimum of 5,000 events in the relevant gate was collected for data analysis using FlowJo version 10.0.6 (TreeStar Inc., Switzerland)

**Uptake of Dil-acetylated low-density lipoprotein**

Confluent monolayers of CEO cells (passage 3) were incubated with 1,1′-dioctadecyl-3,3,3′tetramethylindocarbocyanine-labelled acetylated low-density lipoprotein (LDL) (Cat no. L-3484; Life Technologies, UK) (1μg/ml) for 4 hours at 37°C (n=3). Samples were then washed and visualised with a fluorescent Zeiss Axio Observer microscope (Zeiss, Germany).

**Western blot**

Cell protein extracts obtained from fresh cells (passage 4) and treated with RIPA buffer supplemented with protease inhibitor cocktail, were electrophoresed on 10% SDS-PAGE and transferred by a wet-transfer method. 30µg of protein were loaded and separated by 10% SDS-PAGE and proteins were transferred to polyvinylidene fluoride (PVDF) membranes, successively incubated with mouse anti-human VEGF (Sc-7269 SantaCruz) primary antibody followed by rabbit anti-mouse P0260 (Dako) secondary antibody. Finally, the blots were treated with the Immobilon ECL Western Blotting Detection Kit Reagent (Millipore) and developed after exposure to Fujifilm X-ray film in the darkroom with chemoluminescence system.

**Griess assay**

Nitrite concentrations were measured by Griess Reagent (Cat no. ab234044, Abcam, UK) assay following the manufacturer´s instructions. Briefly 1x10^6^ endothelial cells (passage 4-6) were lysed by 100μl ice cold Nitrite Assay Buffer and kept on ice for 10 minutes followed by centrifugation at 10.000xg for 5 minutes. Supernatant was then transferred to a 96-well plate and incubated 10 minutes at RT with the reaction mix next to calibration curve dilutions in duplicate. Absorbance is measured at 540 nm in end-point mode at RT. Nitrite concentration (nmol/well) was then calculated from the standard curve and corrected by sample volume added in the sample wells.

**Functional characterisation of endothelial cells**

**Growth kinetics**

At second and subsequent passages, seeding density (C*_s_*), days in culture (*t*) and harvested cell number (C*_h_*) were recorded. Cells were plated at a concentration of 1x10^4^ in a 12-well collagen I coated plate in 1ml of EGM-2 endothelial media (Lonza). At each subsequent passage, the cells were counted and plated again at the same cell concentration. Population doubling time (PDT) was calculated according to the equation: PDT= Log^2^(C*_h_*/C*_s_*)/*t* as previously described [1]. Cumulative population doubling levels (CPDL) (the sum of all population doublings) were also calculated.

**Glucose consumption**

Glucose concentration was determined by spectrophotometry (COBAS Mira Plus, Horiba ABX) from cell culture media (48 hours) (cells passage 5) by monitoring the production of NAD(P)H in the specific reaction at 340 nm wavelength. More specifically, glucose concentration was measured using hexokinase (HK) and glucose-6-phosphate dehydrogenase (G6PDH) coupled with enzymatic reactions (commercial enzymatic kit). The metabolite consumption normalized rates resulted from the concentrations of the measured metabolite and corrected according to cell proliferation under exponential growth conditions. All the values are expressed in micromol or nanomol of metabolite consumed or produced per hour and 10^6^ cells (µmol/h·10^6^ cells or nmol/h·10^6^cells) and represented as a percentage of CEO cells glucose consumption with respect to the HCAECs control.

**Adhesion assay**

Endothelial cells (passage 3) were trypsinised from tissue culture plastics and aliquots of 4x10^4^ cells were resuspended in 1ml EGM™-2 medium and seeded into 1 well of a BD BioCoat Collagen I coated 6 well plate for 30 minutes. Wells were gently washed to remove non-adherent cells and mosaics of digital photographs across the centre of the well were captured. Attachment within a defined region of each well was quantified and expressed as a percentage of seeded cell number [4].

**Wound migration assay**

Endothelial cells (passage 3) were grown to 90% confluence and rendered quiescent by incubation with serum-free EGM™-2 medium for 24 hours at 37°C. A linear vertical ‘wound’ was created across the diameter of the 6 well plate using a sterile P1000 pipette tip. Cells were then washed and replenished with serum-free EGM™-2 medium. Sets of digital images were taken at 0 hours and 24 hours. To quantify migration, the width of the wound was visualised at the start time (0 hours) and the area of wound closure across the vertical stroke at 24 hours was quantified and expressed as a percentage of wound closure^4^.

**Angiogenesis assay**

BD Matrigel™ basement membrane matrix (Cat no. 365231; BD Pharmingen, UK) was thawed on ice and 150μl was laid into a Corning® Costar® 48 well, flat bottom cell culture plates (Cat. CLS3548; Sigma Aldrich, UK) and incubated for 30 minutes at 37°C. After gelification, 2 x10^4^ cells (passage 3) in 100μl EGM™-2 medium supplemented with 10% HyClone™ FBS were seeded onto the Matrigel™ . After 24 hours, mosaics of digital images across the entire well were captured. Tubule formation was quantified as the number of complete tubule structures present in the 48 well plate.

**Subcutaneous sponge implantation assay for *in vivo* angiogenesis**

Male NOD-SCID gamma mice (NOD.Cg-PrkdcscidIl2rgtm1WjI/SzJ) (Stock no. 005557; Charles River, UK) aged 10-12 weeks (CEO cells and EOCs n=12 each, 3 biological repeats; HUVECs n=4, 1 biological repeat) were purchased and maintained in the Biomedical research facility at Edinburgh University. All animal experiments were carried out in accordance with the British Home Office Animals (Scientific Procedures) Act 1986 in accordance with institutional guidelines.

Endothelial cells (passage 3) were trypsinised from tissue culture vessels and aliquots of 1 x 10^5^ cells were resuspended in 100µl 1:1 EGM™-2/phenol free GFR Matrigel™ (Cat 356231; BD Biosciences, UK). Sterilised sponge cylinders (0.5cm/1cm) (Caligen Foam, U.K) were compressed into this suspension and incubated for 30 minutes at 37°C. EGM™-2/phenol free GFR Matrigel™ sponges were prepared as matched controls. Mice were anaesthetised intraperitoneally with Domitor containing Medetomidine and Vetalar containing Ketamine; both were administered at 0.1ml per 10g body weight. Upon sedation, an analgesic, Vetergesic containing buprenorphine was administered subcutaneously at 0.1mg per kg body weight. Matrigel™ sponges (vehicle control) were implanted subcutaneously into the left flank and cell-embedded sponges were implanted in the right flank [5]. The sides of the wound were apposed with a forceps and closed using skin staples (9mm Autoclips; Cat no. NC9050532; Fisher Scientific, USA). After surgery, a reversal agent, Antisedan-containing Atipamezole, was administered subcutaneously at 0.05ml per 10g body weight and the mice observed on a heat mat until recovery. The wound was observed daily throughout the experiment and after 7 days the staples were removed. After 21 days, mice were sacrificed by cervical dislocation and the sponges removed. Sponges were fixed in 4% PFA overnight, then transferred to 70% ethanol and stored at 4°C.

**Chalkley count**

Fixed sponges were paraffin-embedded and cut transversely into 4µm section. Sections were deparaffinised, rehydrated and stained with haematoxylin and eosin as before. Vessel density within sponges was quantified using the Chalkley count [2,5-6]. The three most vascular areas (hot spots) with the highest number of microvessel profiles were chosen subjectively from each slide (3 slides per sponge per animal). Using a x20 objective, a 25-point Chalkley eyepiece graticule (NG52 Chalkley Point Array 26mm; Cat no. 01B26257; Pyser-SGI Ltd, UK) was applied to each hot-spot area and oriented to permit the maximum number of dot-perfusing vessel intersections (which were defined by the presence of red blood cells). The Chalkley score was determined by the sum of points coinciding with a vessel.

**Immunohistochemistry**

The presence of human cells within murine vessels was determined using human specific monoclonal antibodies to endothelial antigens [5]. Paraffin embedded 4µm sections of sponges were deparaffinised and rehydrated prior to antigen retrieval with Tris based buffer at 100°C for 20 minutes. Specimens were cooled for 40 minutes at room temperature, then washed in PBS, permeablised and incubated with 10% NGS as before. Specimens were incubated overnight at 4°C with monoclonal antibodies: human specific mouse anti-CD146 and cross-reactive rabbit anti-CD31 (**Online Table 1**). Specimens were washed and incubated with fluorescent-labelled secondary antibodies: Alexa Fluor® 488 goat anti-mouse and Alexa Fluor® 568 goat anti-rabbit to detect CD146 and CD31, respectively (**Online Table 1**). Slides were then rinsed in tap water and mounted as before. The EGM™-2/ phenol free GFR Matrigel™ -only sponges were used as negative control for human cells. Human fibroid tissue was used as a positive human control for primary antibody immunoreactivity. Using a x20 objective, three hotspot regions were chosen subjectively from each slide based on visual scanning for cross-reactive CD31 positive vessels (red). Composite multicolour images of each region were generated, CD31-positive and human-specific CD146-positive vessels (green) were counted, and the percentage of CD146-positive vessels was calculated.

**Image analysis**

Carstairs histological images were captured using the Olympus Provis AX-70 microscope (Olympus, USA). All other images were captured using a fluorescent Zeiss Axio Observer microscope (Zeiss, Germany). All images were processed using AxioVision 4.8 software (Carl Zeiss, Germany) and Photoshop version CS5 (Adobe, USA) was used when image quantification was required.

**Statistical analysis**

Data are shown as mean ± standard deviation. Independent samples were analysed using the unpaired Student’s *t*-test. More than two groups were compared using repeated measure or one-way analysis of variance (ANOVA) with Bonferroni post-tests where appropriate. Categorical data were compared using the chi-squared with Fisher’s exact test. Paired Student’s *t*-tests were used to compare Chalkley counts between vehicle control- and cell-infiltrated sponges. Statistical significance was assumed if a null hypothesis could be rejected at P≤ 0.05.

**References**

[1]. Ingram DA, Mead LE, Tanaka H, Meade V, Fenoglio A, Mortell K, Pollok K, Ferkowicz MJ, Gilley D, Yoder MC. Identification of a novel hierarchy of endothelial progenitor cells using human peripheral and umbilical cord blood. *Blood*. 2004;104:2752-2760

[2]. Tura O, Skinner EM, Barclay GR, Samuel K, Gallagher RC, Brittan M, Hadoke PW, Newby DE, Turner ML, Mills NL. Late outgrowth endothelial cells resemble mature endothelial cells and are not derived from bone marrow. *Stem cells*. 2013;31:338-348

[3]. von Bruhl ML, Stark K, Steinhart A, Chandraratne S, Konrad I, Lorenz M, Khandoga A, Tirniceriu A, Coletti R, Kollnberger M, Byrne RA, Laitinen I, Walch A, Brill A, Pfeiler S, Manukyan D, Braun S, Lange P, Riegger J, Ware J, Eckart A, Haidari S, Rudelius M, Schulz C, Echtler K, Brinkmann V, Schwaiger M, Preissner KT, Wagner DD, Mackman N, Engelmann B, Massberg S. Monocytes, neutrophils, and platelets cooperate to initiate and propagate venous thrombosis in mice in vivo. *The Journal of experimental medicine*. 2012;209:819-835

[4]. Reinhart‐King CA. Endothelial cell adhesion and migration. *Methods in enzymology*. 2008;443:45-64

[5]. Barclay GR, Tura O, Samuel K, Hadoke PW, Mills NL, Newby DE, Turner ML. Systematic assessment in an animal model of the angiogenic potential of different human cell sources for therapeutic revascularization. *Stem cell research & therapy*. 2012;3:23

[6]. Chalkley HW. Method for the quantitative morphologic analysis of tissues. 1943

7. Bardin N, Anfosso F, Massé J-M, Cramer E, Sabatier F, Le Bivic A, Sampol J, Dignat-George F. Identification of cd146 as a component of the endothelial junction involved in the control of cell-cell cohesion. *Blood*. 2001;98:3677-3684

8. Osawa M, Masuda M, Harada N, Lopes RB, Fujiwara K. Tyrosine phosphorylation of platelet endothelial cell adhesion molecule-1 (pecam-1, cd31) in mechanically stimulated vascular endothelial cells. *European journal of cell biology*. 1997;72:229-237

9. Cheifetz S, Bellon T, Cales C, Vera S, Bernabeu C, Massague J, Letarte M. Endoglin is a component of the transforming growth factor-beta receptor system in human endothelial cells. *Journal of Biological Chemistry*. 1992;267:19027-19030

10. Kim I, Moon S-O, Kim SH, Kim HJ, Koh YS, Koh GY. Vascular endothelial growth factor expression of intercellular adhesion molecule 1 (icam-1), vascular cell adhesion molecule 1 (vcam-1), and e-selectin through nuclear factor-κb activation in endothelial cells. *Journal of Biological Chemistry*. 2001;276:7614-7620

11. Gavard J, Gutkind JS. Vegf controls endothelial-cell permeability by promoting the β-arrestin-dependent endocytosis of ve-cadherin. *Nature cell biology*. 2006;8:1223-1234

12. Fina L, Molgaard HV, Robertson D, Bradley NJ, Monaghan P, Delia D, Sutherland DR, Baker MA, Greaves MF. Expression of the cd34 gene in vascular endothelial cells. *Blood*. 1990;75:2417-2426

13. Terman BI, Dougher-Vermazen M, Carrion ME, Dimitrov D, Armellino DC, Gospodarowicz D, Böhlen P. Identification of the kdr tyrosine kinase as a receptor for vascular endothelial cell growth factor. *Biochemical and biophysical research communications*. 1992;187:1579-1586

14. Shmelkov SV, Clair RS, Lyden D, Rafii S. Ac133/cd133/prominin-1. *The international journal of biochemistry & cell biology*. 2005;37:715-719

**Online Figures Legends**

**
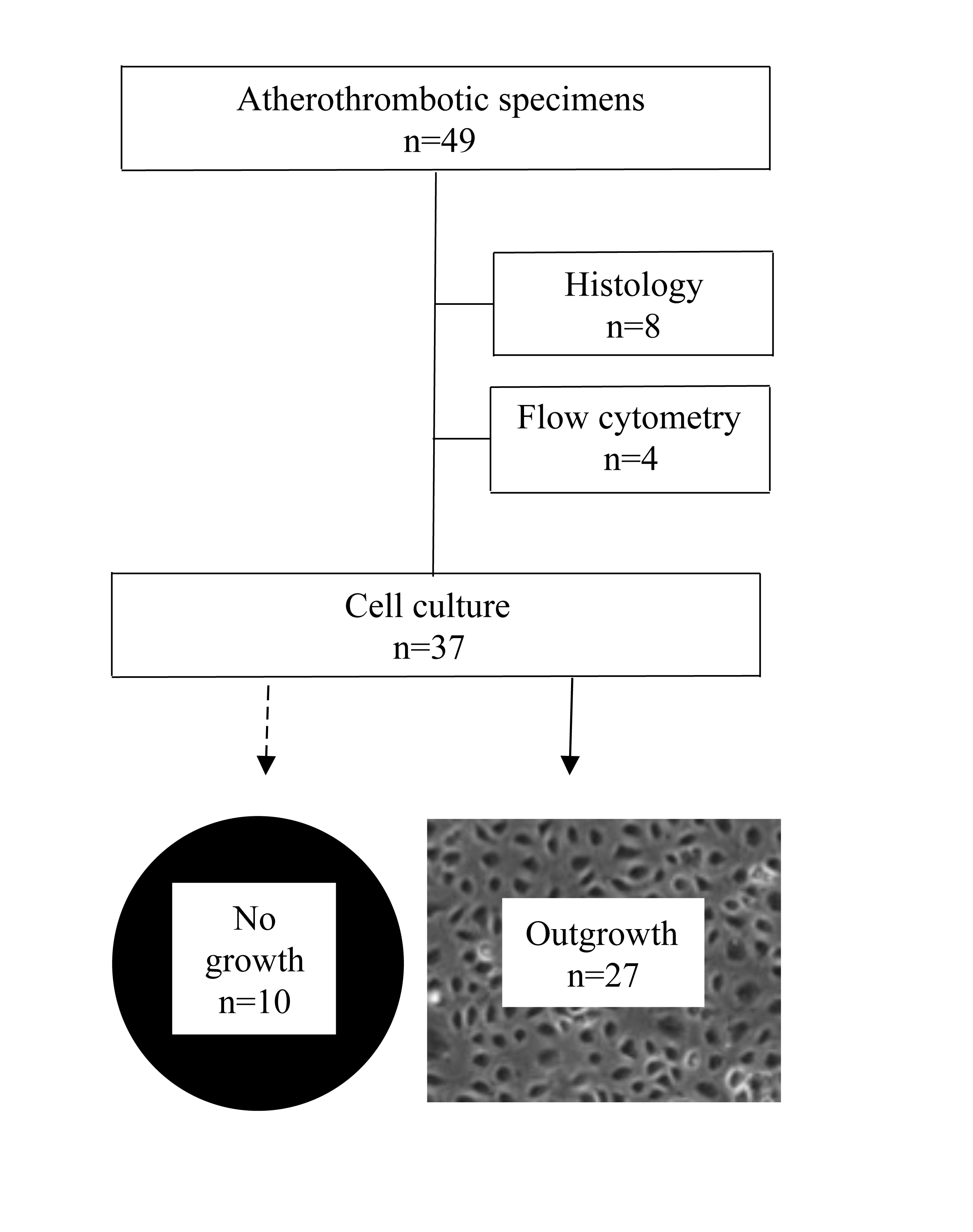
**

**Online Fig. 1: Atherothrombotic specimen allocation.**

Atherothrombotic specimens were fixed for histological (n=8) and flow cytometric (n=4) examination and were physically disaggregated for cell culture (n=37). 27/37 (73%) of atherothrombotic specimens gave rise to coronary endothelial outgrowth.

**
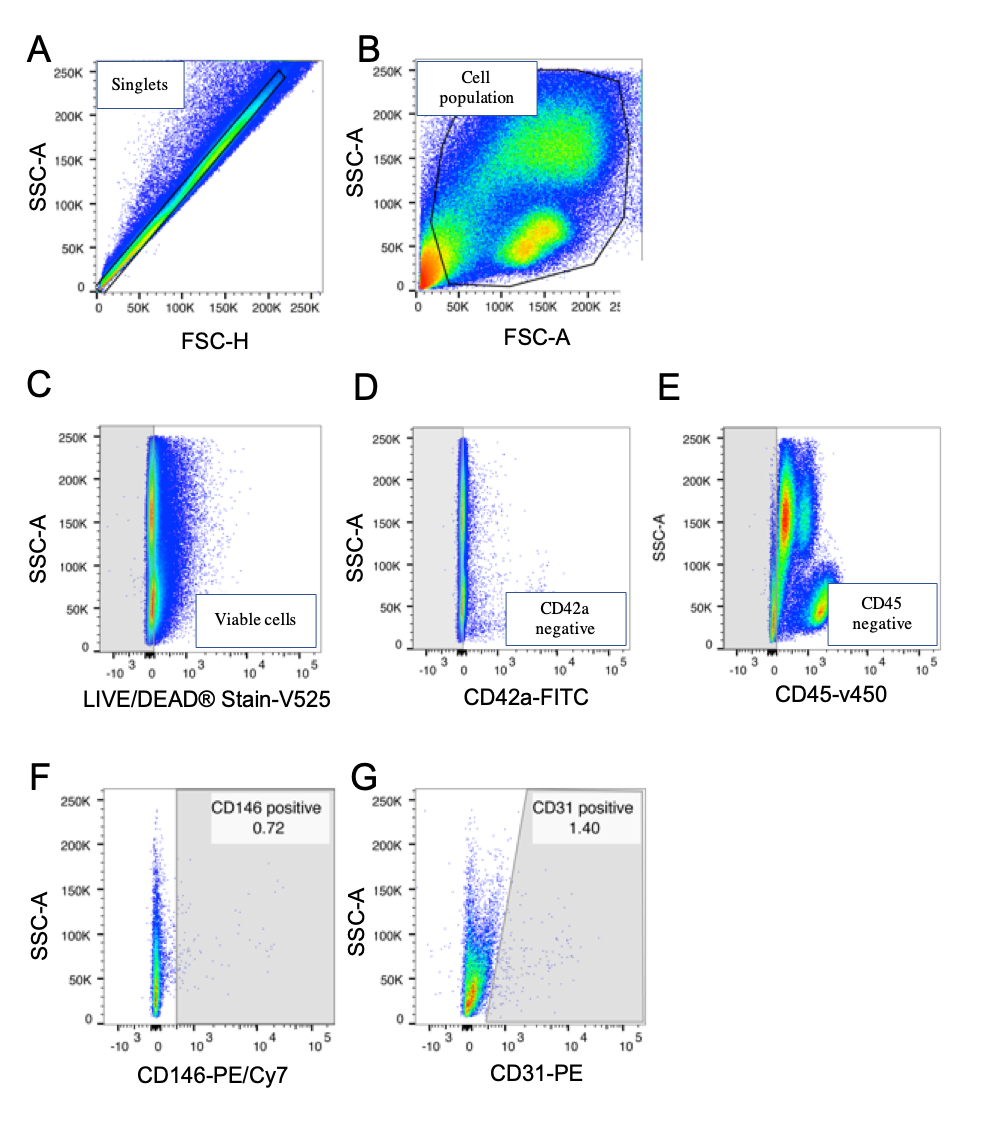
**

**Online Fig. 2: Representative figure for flow cytometric analysis of atherothrombotic specimens.**

Gating strategy for the identification of viable CD42a^-^CD45^-^CD146^+^ and CD42a^-^CD45^-^CD31^+^ cells within atherothrombotic specimens. Following doublet discrimination (***A***) and scatter selection (***B***), viable cells were selected by exclusion of LIVE/DEAD® Fixable Dead Cell Stain-V525 (***C***). CD42a (***D***) and CD45 (***E***) negative cells were selected to avoid contamination by platelets, hematopoietic cells and leucocytes. Endothelial cells were identified by expression CD146 (***F***) or CD31 (***G***).

**Online Fig. 3: Flow cytometric dot-plot analysis of αSMA^+^ in coronary endothelial outgrowth cells.**

Relative size (FCS-A axis) and granularity (SSC-A axis) (***A***), unstained sample (***B)*** and sample incubated against anti-α smooth muscle actin antibody ***(C)***.

**Online Fig. 4: Glucose consumption, VEGF protein levels and nitrite concentration in coronary endothelial outgrowth cells compared to control cells.** Metabolic measurement of glucose consumption ***(A).*** Data are represented as rate (%) of CEO cells glucose consumption respect to the Human Coronary Artery Endothelial Cells (HCAECs) control. The graph shows the average of the glucose consumption of n=8 CEO cell lines. The bars correspond to the mean ± SD of n=3 independent experiments. Statistically significant differences between CEO cells and HCAECs control cells were indicated by Student’s t-test * p < 0,05 ***(A).*** Protein levels of VEGF in CEO cells and HCAECs. Protein band quantification was performed by Image-J program. Each band has been quantified and compared to β-actin as a loading control. Data represented as mean ± SD of n=3 independent experiments, Student’s t-test, p >0,05 ***(B).*** Nitrite concentration (mM) was measured by the Griess reagent assay (ab234044) as an indirect measure of nitric oxide (NO) production. Data represented as mean ± SD, n=3 ***(C).***

**Online Tables**

**Online Table 1:** Suppliers of primary and secondary antibodies for immunocytochemistry.

|  |  |  |  |  |  |
| --- | --- | --- | --- | --- | --- |
| **Antibody** | **Raised in** | **Reactivity** | **Supplier** | **Catalogue no.** | **Concentration** |
| **Primary** |  |  |  |  |  |
| CD31 | Rabbit | Mouse/Human | Abcam | ab28364 | 1:100 |
| vWF | Mouse | Human | BD Pharmingen | 555849 | 1:250 |
| CD146 | Mouse | Human | Abcam | ab49492 | 1:50 |
| **Secondary** |  |  |  |  |  |
| Biotinylated anti-mouse IgG | Horse | Mouse | Vector Laboratories | BA-2000 | 1:250 |
| AF488 | Goat | Rabbit | Life Technologies | A11034 | 1:200 |
| AF647 | Goat | Mouse | Life Technologies | A21240 | 1:200 |
| AF488 | Goat | Mouse | Life Technologies | A11001 | 1:200 |
| AF568 | Goat | Rabbit | Life Technologies | A11011 | 1:200 |
|  |  |  |  |  |  |

vWF= von Willebrand factor.

**Online Table 2:** Antigens routinely used to identify endothelial cells.

eNOS= endothelial nitric oxide synthase; HPCA= hematopoietic progenitor cell antigen; ICAM= intracellular adhesion molecule; KDR=  kinase domain receptor; MCAM= melanoma cell adhesion molecule; PECAM= platelet endothelial cell adhesion molecule; VCAM= vascular cell adhesion molecule; VE= vascular endothelial; vWF= von Willebrand factor.

**Online Table 3**: Suppliers of antibodies for flow cytometry.

|  |  |  |  |  |
| --- | --- | --- | --- | --- |
| **Antibody** | **Fluorochrome** | **Supplier** | **Catalogue no.** | **Concentration** |
| **Cell phenotyping** | |  |  |  |
| **Panel I** |  |  |  |  |
| CD31 | FITC | BD Biosciences | 555445 | 1:100 |
| CD146 | Pe/Cy7 | Biolegend | 342010 | 1:100 |
| CD34 | APC/Cy7 | Biolegend | 343514 | 1:100 |
| CD309 | PE | R and D systems | P35968 | 1:50 |
| CD45 | V450 | BD Horizon | 560368 | 1:100 |
| CD133 | APC | Miltenyl Biotec | 130-090-826 | 1:50 |
| **Panel II** |  |  |  |  |
| CD54 | PE | BD Pharmingen | 555511 | 1:100 |
| CD106 | FITC | BD Biosciences | 551146 | 1:100 |
| **Panel III** |  |  |  |  |
| CD105 | APC | R and D systems | FAB10971A | 1:100 |
| CD144 | PE | R and D systems | FAB9381P | 1:100 |
| **Panel IV** |  |  |  |  |
| CD117 | PE | R and D systems | FAB322F | 1:50 |
| **Panel V** |  |  |  |  |
| vWF | FITC | BD Biosciences | 5601013 | 1:200 |
| eNOS | PE | BD Biosciences | 560103 | 1:100 |
| **Panel VI** |  |  |  |  |
| αSMA | AF405 | Abcam | ab210128 | 1:50 |
| **Atherothrombotic cell analysis** | | |  |  |
| LIVE/DEAD® Fixable Dead Cell Stain | Aqua | Life Technologies | L34957 | 1:100 |
| CD45 | V450 | BD Horizon | 560368 | 1:100 |
| CD31 | PE | Biolegend | 303106 | 1:50 |
| CD146 | Pe/Cy7 | Biolegend | 342010 | 1:100 |
| CD42a | FITC | BD Pharmingen | 558818 | 1:100 |
|  |  |  |  |  |

APC= Allophycocyanin; eNOS= endothelial nitric oxide synthase; FITC= fluorescein isothiocyanate; PE= Phycoerythrin; vWF= von Willebrand factor.

**Online Table 4:** Clinical and procedural characteristics of all patients undergoing thrombectomy for ST-segment myocardial infarction

|  |  |
| --- | --- |
|  | **Total population *(n=49*)** |
| Age, years | 62 ± 12 |
| Gender, male | 37 (76%) |
| **Medical history and risk factors** |  |
| Previous myocardial infarction | 5 (10%) |
| Previous PCI/CABG | 6 (12%) |
| Current smoker | 21 (43%) |
| Ex-smoker | 4 (8%) |
| Hypertension | 10 (20%) |
| Hyperlipidaemia | 13 (27%) |
| Family history of premature coronary heart disease | 17 (35%) |
| Diabetes mellitus | 6 (12%) |
| **Medication on admission** |  |
| Aspirin | 7 (14%) |
| Clopidogrel | 3 (6%) |
| B-blockers | 4 (8%) |
| ACE-inhibitors | 8 (16%) |
| Statins | 10 (20%) |
| **Acute coronary syndrome** |  |
| Troponin I concentration, micrograms/L | 32.3 ± 18.8 |
| **Culprit vessel** |  |
| Left anterior descending artery | 15 (31%) |
| Circumflex artery | 3 (6%) |
| Right coronary artery | 31 (63%) |

Values are number (%) or mean ± standard deviation. ACE = angiotensin converting enzyme; CABG = coronary artery bypass grafting; PCI = percutaneous coronary intervention.

**Online Table 5.** Clinical characteristics of patients with and without coronary endothelial outgrowth following thrombectomy for ST-segment elevation myocardial infarction.

|  | **Total population (*n=37*)** | | |
| --- | --- | --- | --- |
|  | **Outgrowth** | **No outgrowth** | **P-value** |
|  | ***n=27*** | ***n=10*** |  |
| Age, years | 60 ±11 | 61 ±11 | 0.885 |
| Gender, male | 19 (70%) | 7 (70%) | 1.000 |
| **Medical history and risk factors** |  |  |  |
| Previous myocardial infarction | 4 (15%) | 1 (10%) | 1.000 |
| Previous PCI/CABG | 4 (15%) | 0 (0%) | 1.000 |
| Current smoker | 14 (52%) | 3 (30%) | 0.287 |
| Ex-smoker | 3 (11%) | 1 (10%) | 1.000 |
| Hypertension | 4 (15%) | 1 (10%) | 1.000 |
| Hyperlipidaemia | 7 (26%) | 2 (20%) | 1.000 |
| Family history of premature coronary heart disease | 13 (48%) | 2 (20%) | 0.153 |
| Diabetes mellitus | 2 (7%) | 1 (10%) | 1.000 |
| **Medication on admission** |  |  |  |
| Aspirin | 5 (19%) | 1 (10%) | 1.000 |
| Clopidogrel | 2 (7%) | 0 (0%) | 1.000 |
| B-Blockers | 3 (11%) | 0 (0%) | 0.548 |
| ACE-Inhibitors | 4 (15%) | 2 (20%) | 0.653 |
| Statins | 7 (26%) | 2 (20%) | 1.000 |
| **Myocardial injury** |  |  |  |
| Troponin I concentration, micrograms/L | 27.9 ± 20.0 | 30.3 ± 14.9 | 0.749 |
| **Culprit vessel** |  |  |  |
| Left anterior descending artery, n=12 | 4 | 8 | - |
| Circumflex artery, n=2 | 2 | 0 | - |
| Right coronary artery, n=23 | 21 | 2 | - |

Values are number (%) or mean±standard deviation. ACE = angiotensin converting enzyme; CABG =coronary artery bypass grafting; PCI = percutaneous coronary intervention.

**Online Table 6.** Phenotypic characterisation of coronary endothelial outgrowth cells throughout culture.

|  |  |  |  |  |  |  |  |
| --- | --- | --- | --- | --- | --- | --- | --- |
| **Antigen** | **Alternative name** | **Passage 1-2** | **Passage 3-4** | **Passage 5-6** | **Passage 7-8** | **Passage 9-10** | **P-value** |
| **Cell surface** |  |  |  |  |  |  |  |
| CD146 | MCAM | 94.5 ± 5.7 | 92.6 ± 12.8 | 88.8 ± 13.4 | 96.7 ± 5.0 | 88.9 ± 15.1 | 0.700 |
| CD31 | PECAM-1 | 86.7 ± 14.4 | 87.4 ± 14.7 | 92.2 ± 11.9 | 94.8 ± 6.1 | 87.5 ± 14.9 | 0.534 |
| CD105 | Endoglin | 92.7 ± 5.8 | 94.1 ± 6.0 | 99.6 ± 0.2 | 99.4 ± 0.3 | 99.7 ± 0.2 | **0.012** |
| CD54 | ICAM-1 | 78.0 ± 36.7 | 84.6 ± 11.3 | 80.3 ± 11.5 | 68.6 ± 9.2 | 86.5 ± 16.8 | 0.646 |
| CD144 | VE-Cadherin | 53.3 ± 39.4 | 49.1 ± 39.4 | 55.8 ± 31.3 | 52.7 ± 24.5 | 56.4 ± 36.4 | 0.998 |
| CD106 | VCAM-1 | 1.1 ± 0.5 | 3.0 ± 5.5 | 2.9 ± 4.0 | 3.7 ± 3.6 | 1.1 ± 0.4 | 0.653 |
| CD34 | HPCA-1 | 59.0 ± 34.3 | 51.5 ± 34.3 | 38.2 ± 28.4 | 29.1 ± 28.5 | 27.3 ± 24.9 | **0.030** |
| CD309 | KDR | 74.4 ± 17.5 | 78.4 ± 22.1 | 90.8 ± 8.8 | 87.2 ± 26.1 | 97.2 ± 2.8 | 0.220 |
| CD133 | Prominin-1 | 43.3 ± 37.7 | 27.0 ± 32.9 | 19.8 ± 32.2 | 3.7 ± 3.1 | 0.8 ± 0.4 | 0.052 |
| CD117 | c-Kit | 2.1 ± 1.3 | 2.5 ± 1.3 | 2.7 ± 1.4 | 2.3 ± 2.2 | 2.4 ± 2.2 | 0.992 |
| **Cytoplasmic** | |  |  |  |  |  |  |
| vWF | - | 99.8 ± 0.5 | 100.0 ± 0.0 | 99.8 ± 0.5 | 100 ± 0.0 | 100.0 ± 0.0 | 0.443 |
| eNOS | - | 74.3 ± 24.2 | 68.5 ± 41.5 | 70.4 ± 35.1 | 90.7 ± 13.2 | 69.6 ± 27.5 | 0.738 |
| **Leucocyte** |  |  |  |  |  |  |  |
| CD45 | PTPRC or LCA | 1.2 ± 0.7 | 1.0 ± 0.5 | 2.2 ± 1.5 | 1.6 ± 0.7 | 1.3 ± 0.6 | 0.237 |

Values are mean ± standard deviation (n=6-10).CEO= coronary endothelial outgrowth; eNOS= endothelial nitric oxide synthase; HPCA= hematopoietic progenitor cell antigen; ICAM= intracellular adhesion molecule; KDR= kinase domain receptor; MCAM= melanoma cell adhesion molecule; LCA= leucocyte common antigen; PECAM= platelet endothelial cell adhesion molecule;PTPRC= protein tyrosine phosphatase receptor type C; VCAM= vascular cell adhesion molecule; VE= vascular endothelial; vWF= von Willebrand factor. One-way analysis of variance (ANOVA).

**Online Table 7.** Coronary endothelial outgrowth phenotype during culture compared with endothelial cells derived from the peripheral circulation (late endothelial outgrowth cells), the coronary arteries and the umbilical veins.

|  | |  |  |  |  |  |  |  |  |  |  |
| --- | --- | --- | --- | --- | --- | --- | --- | --- | --- | --- | --- |
| **Antigen** | **Early passage (1-4)** | | | | |  | **Late passage (9-12)** | | | | |
|  | **CEO cells** | **EOCs** | **HCAECs** | **HUVECs** | **P-Value** |  | **CEO cells** | **EOCs** | **HCAECs** | **HUVECs** | **P-Value** |
|  | ***n=8*** | ***n=3*** | ***n=3*** | ***n=3*** |  |  | ***n=8*** | ***n=3*** | ***n=3*** | ***n=3*** |  |
| **Cell surface** |  |  |  |  |  |  |  |  |  |  |  |
| CD146 | 93.4 ± 10.3 | 90.3 ± 6.8 | 99.1 ± 0.2 | 97.4 ± 1.0 | 0.839 |  | 93.0 ± 12.5 | 96.0 ± 3.6 | 93.7 ± 0.2 | 98.0 ± 2.4 | 0.470 |
| CD31 | 87.1 ± 14.0 | 95.7 ± 2.0 | 95.2 ± 8.1 | 94.1 ± 5.0 | 0.230 |  | 88.9 ± 14.7 | 81.5 ± 9.6 | 89.7 ± 1.1 | 94.0 ± 6.0 | 0.555 |
| CD105 | 93.4 ± 5.5 | 96.2 ± 5.7 | 99.3 ± 0.3 | 98.9 ± 1.0 | 0.353 |  | 99.5 ± 0.6 | 99.4 ± 0.5 | 99.0 ± 0.3 | 99.5 ± 0.4 | 0.953 |
| CD54 | 81.8 ± 23.9 | 92.0 ± 13.0 | 97.2 ± 0.3 | 95.1 ± 6.3 | 0.085 |  | 84.6 ± 16.1 | 79.1 ± 9.4 | 70.7 ± 1.5 | 85.9 ± 12.1 | 0.443 |
| CD144 | 51.2 ± 36.5 | 60.9 ± 36.2 | 37.9 ± 30.7 | 75.7 ± 29.3 | 0.432 |  | 48.9 ± 38.7 | 16.9 ± 19.3 | 2.7 ± 0.8 | 68.0 ± 52.1 | 0.132 |
| CD34 | 63.5 ± 24.5 | 43.7 ± 9.4 | 98.0 ± 0.3 | 48.9 ± 12.5 | **0.017** |  | 35.3 ± 25.6 | 49.8 ± 37.0 | 98.5 ± 1.5* | 36.8 ± 35.6 | **0.010** |
| CD309 | 76.8 ± 19.8 | 80.0 ± 31.0 | 42.6 ± 28.4 | 92.1 ± 10.8 | **0.046** |  | 89.0 ± 21.4 | 70.3 ± 23.1 | 6.5 ± 1.2*** | 84.6 ± 25.7 | **0.001** |
| CD133 | 33.5 ± 34.6 | 3.0 ± 1.9 | 0.1 ± 0.1 | 10.1 ± 15.0 | 0.172 |  | 1.6 ± 1.3 | 1.6 ± 1.9 | 0.1 ± 0.1*** | 1.1 ± 0.3 | **0.001** |
| CD117 | 2.3 ± 1.2 | 2.3 ± 1.0 | 0.6 ± 0.3 | 2.0 ± 0.4 | 0.165 |  | 2.5 ± 2.1 | 3.4 ± 5.2 | 0.1 ± 0.1 | 4.8 ± 1.5 | **0.006** |
| CD106 | 2.2 ± 4.2 | 1.1 ± 0.3 | 1.7 ± 0.3 | 1.3 ± 0.7 | 0.967 |  | 1.1 ± 0.4 | 2.1 ± 2.1 | 0.6 ± 0.1 | 1.3 ± 0.9 | 0.316 |
| **Cytoplasmic** |  |  |  |  |  |  |  |  |  |  |  |
| vWF | 99.9 ± 0.4 | 100 ± 0 | 99.0 ± 0.1 | 100 ± 0 | 0.859 |  | 100 ± 0 | 100 ± 0 | 99.8 ± 0.1 | 100 ± 0 | 0.147 |
| eNOS | 71.4 ± 31.6 | 26.0 ± 39.7 | 37.4 ± 18.0 | 87.3 ± 5.2 | **0.019** |  | 73.9 ± 27.6 | 56.1 ± 27.9 | 20.0 ± 18.9 | 50.9 ± 44.3 | **0.014** |
| **Leucocyte** |  |  |  |  |  |  |  |  |  |  |  |
| CD45 | 1.1 ± 0.6 | 2.6 ± 0.2 | 1.0 ± 0.2** | 1.9 ± 1.1 | **0.005** |  | 1.1 ± 0.6 | 1.3 ± 0.5 | 0.5 ± 1.0 | 1.6 ± 0.2 | **0.035** |

Values are mean ± standard deviation. CEO= coronary endothelial outgrowth; eNOS= endothelial nitric oxide synthase; EOC= endothelial outgrowth cell; HCAECs= human coronary artery endothelial cells; HUVEC= human umbilical vein endothelial cell; vWF= von Willebrand factor.Two-way analysis of variance (ANOVA) with Bonferroni post-test*P<0.05, **P<0.01, ***P<0.001*versus*CEO cell.
